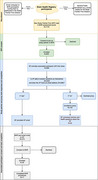# Study Partner First: A new Brain Health Registry initiative to enroll study partners and participants in AD research

**DOI:** 10.1002/alz70860_101361

**Published:** 2025-12-23

**Authors:** Erika Cavallone, Miriam T. Ashford, Joseph Eichenbaum, Derek Flenniken, Alexander Happ, Winnie Kwang, Monica R. Camacho, Juliet Fockler, Scott R. Mackin, Diana Truran‐Sacrey, Michael W Weiner, Rachel L. Nosheny

**Affiliations:** ^1^ Northern California Institute for Research and Education (NCIRE), San Francisco, CA, USA; ^2^ San Francisco Veterans Affairs Medical Center, San Francisco, CA, USA; ^3^ University of California, San Francisco, San Francisco, CA, USA; ^4^ VA Advanced Imaging Research Center, San Francisco Veterans Affairs Medical Center, San Francisco, CA, USA; ^5^ Northern California Institute for Research & Education (NCIRE), San Francisco, CA, USA; ^6^ University of California San Francisco (UCSF), San Francisco, CA, USA; ^7^ San Francisco Veterans Administration Medical Center (SFVAMC), San Francisco, CA, CA, USA

## Abstract

**Background:**

Most Alzheimer's disease (AD) and AD related dementia clinical research studies and trials requires study partner enrollment. The participant is usually the first point of contact. We developed and pilot tested a novel approach in an online research registry, which recruits the study partner first.

**Method:**

The Brain Health Registry (BHR) is an online research registry (*n* >106,000), collecting longitudinal self‐reported health and cognitive data. The BHR Study Partner Portal allows BHR participants to enroll a study partner (SP) in BHR. The SP answers questions about the participant's brain health. Since 2016, >10,000 participant‐SP pairs (dyads) have enrolled. In July 2024, BHR launched a novel feature to expand SP data collection. Enrolled BHR participants are presented with a new task, which allows them to enroll as a SP without having been invited by a participant. The SP can either invite an associated participant (AP) to join (if the SP feels the AP is capable of enrolling and has an email address), or the SP can serve as a *proxy* and complete assessments about the AP on their behalf. Here we assess the preliminary feasibility of this approach in terms of enrollment and task completion.

**Result:**

Within 7 months, 1,144 participants registered in SP First task. 699 have provided their AP's first name. 553 have indicated that they believed their AP was capable of enrolling, and 508 provided their AP's email address. Out of 473 APs that received an email invitation, 40 APs enrolled, and 17 APs completed all assessments. 380 SPs indicated that their AP either didn’t have an email address and/or was not capable of enrolling. Of those, 103 SPs agreed to serve as a Proxy for their AP, and 52 SPs completed all proxy questionnaires.

**Conclusion:**

Our findings demonstrate feasibility of the approach, which can be used to engage and assess SPs whose AP drops out of a study or cannot enroll, mitigating the selection bias against older adults who are too impaired or unmotivated to participate. Future efforts will expand this initiative through email invitations to BHR participants and recruitment of the public through social media.